# Supplementary material for: The Relationship between Obesity and Clinical Outcomes in Young People with Duchenne Muscular Dystrophy
Source: Nutrients. 2022 Aug 12;14(16):3304. doi: 10.3390/nu14163304 (PMC9412587; doi:10.3390/nu14163304)
Supplement: Supplementary file 1 [file nutrients-14-03304-s001.zip › nutrients-1784862-supplementary.pdf]

Supplementary Data

Supplementary Table S1

BMI Status at Ages Five to Nine Years as A Predictor of Time to Loss of Ambulation Using A Cox Proportional Hazards Model [1]

Age at BMI measure	BMI status	Hazard Ratio	95% CI lower	95% CI upper	P-value
**Five years**	*Overweight*	1.237	0.572	2.675	0.590
	Obese	0.644	0.178	2.338	0.504
**Six years**	*Overweight*	0.602	0.242	1.499	0.276
	Obesity	0.771	0.357	1.662	0.507
**Seven years**	*Overweight*	0.930	0.470	1.839	0.834
	Obese	0.737	0.351	1.546	0.419
**Eight years**	*Overweight*	0.820	0.406	1.658	0.581
	Obese	0.714	0.368	1.383	0.317
**Nine years**	*Overweight*	1.211	0.608	2.410	0.587
	Obese	0.919	0.458	1.844	0.812

[1] *Five years*: event n=30, censored n=45, missing n=80, censored cases before the earliest event in a stratum n=3. *Six years*: event n=37, censored n=49, missing n=71, censored cases before the earliest event in a stratum n=1. *Seven years*: event n=46, censored n=48, missing n=61, excluded loss of ambulation prior to seven years n=3. *Eight years*: event n=52, censored n=41, missing n=56, excluded loss of ambulation prior to eight years n=9. *Nine years*: event n=48, censored n=35, missing n=57, excluded loss of ambulation prior to nine years n=18

Reference category is no overweight or obesity

Supplementary Table S2

BMI Status at Ages Five to Nine Years as a Predictor of Time to a 10m Walk/Run Completed in >10 Seconds Using a Cox Proportional Hazards Model [1]

Age at BMI measure	BMI status	Hazard Ratio	95% CI lower	95% CI upper	P-value
**Five years**	*Overweight*	1.655	0.598	4.581	0.332
	Obese	0.869	0.179	4.219	0.862
**Six years**	*Overweight*	0.849	0.261	2.763	0.786
	Obese	1.236	0.420	3.633	0.700
**Seven years**	*Overweight*	1.451	0.624	3.375	0.387
	Obese	1.302	0.503	3.373	0.587
**Eight years**	*Overweight*	0.648	0.251	1.675	0.371
	Obese	0.776	0.330	1.826	0.561
**Nine years**	*Overweight*	1.561	0.614	3.967	0.349
	Obese	1.363	0.549	3.386	0.505

[1] *Five years*: event n=17, censored n=31, missing n=103, censored cases before the earliest event in a stratum n=7. *Six years*: event n=19, censored n=42, missing n=97. *Seven years*: event n=29, censored n=43, missing n=86. *Eight years*: event n=30, censored n=36, missing n=90, excluded10m walk/run completed in >10 seconds occurred prior to eight years n=2. *Nine years*: event n=28, censored n=30, missing n=95, excluded 10m walk/run completed in >10 seconds occurred prior to nine years n=5

Reference category is no overweight or obesity

Supplementary Table S3

BMI Status at Ages Five to Nine Years as a Predictor of Time to a 10m Walk/Run Completed in 7-10 Seconds Using a Cox Proportional Hazards Model [1]

Age at BMI measure	BMI status	Hazard Ratio	95% CI lower	95% CI upper	P-value
**Five years**	*Overweight*	0.935	0.447	1.954	0.857
	Obese	0.440	0.100	1.941	0.278
**Six years**	*Overweight*	0.913	0.405	2.058	0.826
	Obese	0.729	0.304	1.749	0.479
**Seven years**	*Overweight*	0.997	0.505	1.967	0.993
	Obese	0.702	0.330	1.493	0.359
**Eight years**	*Overweight*	0.486	0.220	1.075	0.075
	Obese	0.428	0.207	0.887	**0.023**
**Nine years**	*Overweight*	0.622	0.249	1.553	0.309
	Obese	0.585	0.275	1.241	0.162

[1] *Five years*: event n=31, censored n=28, missing n=98, censored cases before the earliest event in a stratum n=1. *Six years*: event n=34, censored n=32, missing n=92. *Seven years*: event n=45, censored n=33, missing n=80. *Eight years*: event n=42, censored n=28, missing n=88, excluded 10m walk/run completed in 7-10 seconds occurred prior to eight years n=11. *Nine years*: event n=35, censored n=21, missing n=102, excluded 10m walk/run completed in 7-10 seconds occurred prior to nine years n=20
Reference category if no overweight or obesity

Supplementary Table S4

BMI Status at Ages Five to Nine Years of as a Predictor of a Four Stair Climb Completed in >8 Seconds Using a Cox Proportional Hazards Model [1]

Age at BMI measure	BMI status	Hazard Ratio	95% CI lower	95% CI upper	P-value
**Five years**					
	Overweight	0.515	0.216	1.231	0.136
	Obese	0.537	0.149	1.941	0.343
**Six years**	*Overweight*	0.895	0.357	2.245	0.813
	Obese	0.701	0.269	1.823	0.466
**Seven years**	*Overweight*	0.842	0.404	1.758	0.647
	Obese	0.682	0.291	1.602	0.380
**Eight years**	*Overweight*	0.632	0.279	1.432	0.272
	Obese	0.507	0.225	1.142	0.101
**Nine years**	*Overweight*	0.718	0.279	1.844	0.718
	Obese	0.619	0.263	1.454	0.619

[1] *Five years*: event n=25, censored n=28, missing n=101, censored cases before the earliest event in a stratum n=4. *Six years*: event n=28, censored n=36, missing n=94. *Seven years*: event n=37, censored n=37, missing n=84. *Eight years*: event n=35, censored n=30, missing n=84, excluded four stair climb completed in >8 seconds occurred prior to eight years n=9. *Nine years*: event n=29, censored n=26, missing n=89, excluded four stair climb completed in >8 seconds occurred prior to nine years n=14

Reference category is no overweight or obesity

Supplementary Table S5

BMI Status at Ages Five to Nine Years of a Predictor of a Supine-To-Stand Completed in >7 Seconds Using a Cox Proportional Hazards Model [1]

Age at BMI measure	BMI status	Hazard Ratio	95% CI lower	95% CI upper	P-value
**Five years**	*Overweight*	0.909	0.449	1.843	0.792
	Obese	0.658	0.219	1.976	0.456
**Six years**	*Overweight*	1.075	0.494	2.339	0.855
	Obese	0.770	0.339	1.750	0.532
**Seven years**	*Overweight*	1.198	0.612	2.345	0.599
	Obese	0.776	0.369	1.632	0.504
**Eight years**	*Overweight*	0.723	0.334	1.565	0.410
	Obese	0.620	0.301	1.276	0.194
**Nine years**	*Overweight*	0.885	0.355	2.209	0.794
	Obese	0.638	0.299	1.361	0.245

[1] *Five years*: event n=35, censored n=24, missing n=98, censored cases before the earliest event in a stratum n=1. *Six years*: event n=38 censored n=31, missing n=89. *Seven* years: event n=46, censored n=31, missing n=81. *Eight years*: event n=41, censored n=26, missing n=81, excluded supine-to-stand completed in >7 seconds occurred prior to eight years n=10. *Nine years*: event n=34, censored n=20, missing n=81, excluded supine-to-stand completed in >7 seconds occurred prior to nine years n=23

Reference category is no overweight or obesity

Supplementary Table S6

BMI Status at Ages Five to Nine Years as a Predictor of a NSAA Score ≤ 9 Using a Cox Proportional Hazards Model [1]

Age at BMI measure	BMI status	Hazard Ratio	95% CI lower	95% CI upper	P-value
**Five years**	*Overweight*	1.317	0.505	3.436	0.573
	Obesity	0.751	0.158	3.578	0.719
**Six years**	*Overweight*	1.894	0.694	5.167	0.212
	Obesity	1.516	0.491	4.678	0.469
**Seven years**	*Overweight*	2.082	0.846	5.124	0.110
	Obesity	1.089	0.326	3.642	0.889
**Eight years**	*Overweight*	0.747	0.260	2.147	0.588
	Obesity	1.080	0.450	2.593	0.863
**Nine years**	*Overweight*	1.076	0.386	3.003	0.889
	Obesity	1.461	0.582	3.665	0.419

[1] *Five years*: event n=19, censored n=32, missing n=104, censored cases before the earliest event in a stratum n=3. *Six years*: event n=21 censored n=39, missing n=98. *Seven years*: event n=24, censored n=42, missing n=92. *Eight years*: event n=27, censored n=35, missing n=94, excluded NSAA score ≤ 9 occurred prior to eight years n=2. *Nine years*: event n=27, censored n=29, missing n=98, excluded NSAA score ≤ 9 occurred prior to nine years n=4

Reference category is no overweight or obesity

Supplementary Table S7

BMI Status at Ages Five to Nine Years as a Predictor of a 6MWD <325m Using a Cox Proportional Hazards Model [1]

Age at BMI measure	BMI status	Hazard Ratio	95% CI lower	95% CI upper	P-value
**Five years**	*Overweight*	0.411	0.088	1.912	0.257
	Obesity	0.205	0.021	2.023	0.175
**Six years**	*Overweight*	1.049	0.267	4.122	0.945
	Obesity	0.487	0.091	2.611	0.401
**Seven years**	*Overweight*	0.752	0.230	2.464	0.638
	Obesity	0.523	0.130	2.107	0.362
**Eight years**	*Overweight*	0.864	0.254	2.943	0.815
	Obesity	0.567	0.185	1.742	0.322
**Nine years**	*Overweight*	1.248	0.360	4.333	0.727
	Obesity	0.876	0.293	2.614	0.812

[1] *Five years*: event n=9, censored n=13, missing n=126, censored cases before the earliest event in a stratum n=10. *Six years*: event n=11 censored n=14, missing n=123, censored cases before the earliest event in a stratum n=10. *Seven years*: event n=15, censored n=23, missing n=118, censored cases before the earliest event in a stratum n=2. *Eight years*: event n=17, censored n=24, missing n=117. *Nine years*: event n=17, censored n=17, missing n=122, excluded 6MWD <325m occuured prior to nine years n=2
Reference category is no overweight or obesity

Supplementary Table S8

BMI Status at Ages Five to Nine Years of as a Predictor of First Fracture Using a Cox Proportional Hazards Model (Unadjusted) [1]

Age at BMI measure	BMI status	Hazard Ratio	95% CI lower	95% CI upper	P-value
**Five years**	*Overweight*	0.523	0.241	1.135	0.101
	Obesity	2.197	0.970	4.975	0.059
**Six years**	*Overweight*	2.022	0.930	4.396	0.076
	Obesity	2.082	0.970	4.470	0.060
**Seven years**	*Overweight*	1.256	0.622	2.539	0.525
	Obesity	1.776	0.875	3.605	0.112
**Eight years**	*Overweight*	1.404	0.676	2.915	0.363
	Obesity	1.862	0.927	3.739	0.081
**Nine years**	*Overweight*	0.957	0.437	2.096	0.912
	Obesity	2.050	1.038	4.046	**0.039***

[1] *Five years*: event n=37, censored n=36, missing n=79, censored cases before the earliest event in a stratum n=4, excluded first fracture before five years n=2. *Six years*: event n=40 censored n=44, missing n=71, censored cases before the earliest event in a stratum n=1, excluded first fracture before six years n=2. *Seven years*: event n=47, censored n=46, missing n=60, excluded first fracture before seven years n=5. *Eight years*: event n=47, censored n=39, missing n=58, excluded first fracture before seven years n=13. *Nine years*: event n=45, censored n=37, missing n=57, excluded first fracture prior to nine years n=19 Reference category is no overweight or obesity

Supplementary Table S9

BMI Status at Ages Five to Nine Years as a Predictor of Scoliosis Using a Cox Proportional Hazards Model [1]

Age at BMI measure	BMI status	Hazard Ratio	95% CI lower	95% CI upper	P-value
**Five years**	*Overweight*	0.572	0.167	1.959	0.374
	Obesity	0.536	0.065	4.394	0.561
**Seven years**	*Overweight*	0.310	0.090	1.074	0.065
	Obesity	0.735	0.267	2.025	0.552
**Eight years**	*Overweight*	1.077	0.441	2.630	0.870
	Obesity	0.544	0.181	1.636	0.279
**Nine years**	*Overweight*	0.472	0.165	1.345	0.160
	Obesity	0.828	0.348	1.970	0.669

[1] *Five years*: event n=12, censored n=53, missing n=80, censored cases before the earliest event in a stratum n=13. *Six years*: not able to be performed as coefficients did not converge. *Seven years*: event n=23, censored n=73, missing n=61, censored cases before the earliest event in a stratum n=1. *Eight years*: event n=27, censored n=65, missing n=61, censored cases before the earliest event in a stratum n=3, excluded scoliosis diagnosis before eight years n=2. *Nine years*: event n=26, censored n=65, missing n=61, censored cases before the earliest event in a stratum n=1, excluded scoliosis diagnosis prior to nine years n=5

Reference category is no overweight or obesity

Supplementary Table S10

BMI Status at Ages Five to Nine Years as a Predictor of OSA Using a Cox Proportional Hazards Model [1]

Age at BMI measure	BMI status	Hazard Ratio	95% CI lower	95% CI upper	P-value
**Five years**	*Overweight*	1.212	0.585	2.510	0.605
	Obese	1.257	0.444	3.561	0.667
**Six years**	*Overweight*	2.590	1.175	5.711	**0.018**
	Obese	3.389	1.469	7.815	**0.004**
**Seven years**	*Overweight*	1.476	0.737	2.955	0.272
	Obese	2.163	1.004	4.660	**0.049**
**Eight years**	*Overweight*	0.659	0.281	1.546	0.338
	Obese	2.461	1.245	4.863	**0.010**
**Nine years**	*Overweight*	0.621	0.274	1.411	0.255
	Obese	2.883	1.481	5.612	**0.002**

[1] *Five years*: event n=35, censored n=41, missing n=78, censored cases before the earliest event in a stratum n=1, excluded OSA diagnosis before five years n=3 *Six years*: event n=37, censored n=49, missing n=67, excluded OSA diagnosis before six years n=5. *Seven years*: event n=44, censored n=48, missing n=58, excluded OSA diagnosis before seven years n=8. *Eight years*: event n=45, censored n=46, missing n=57, excluded OSA diagnosis before eight years n=10. *Nine years*: event n=47, censored n=43, missing n=55, excluded OSA diagnosis prior to nine years n=13
Reference category is no overweight or obesity

Supplementary Table S11

BMI Status at Ages Five to Nine Years as a Predictor of CPAP Initiation Using a Cox Proportional Hazards Model [1]

Age at BMI measure	BMI status	Hazard Ratio	95% CI lower	95% CI upper	P-value
**Six years**	*Overweight*	0.528	0.065	4.313	0.551
	Obesity	2.716	0.904	8.155	0.075
**Seven years**	*Overweight*	3.785	1.137	12.598	0.030
	Obesity	3.735	1.053	13.252	**0.041**
**Eight years**	*Overweight*	0.476	0.104	2.177	0.338
	Obesity	2.207	0.824	5.909	0.115
**Nine years**	*Overweight*	0.764	0.248	2.352	0.639
	Obesity	2.117	0.843	5.316	0.110

[1] *Five years*: not able to be performed as coefficients did not converge. *Six years*: event n=14, censored n=54, missing n=70, censored cases before the earliest event in a stratum=20. *Seven years*: event n=18, censored n=64, missing n=61, censored cases before the earliest event in a stratum n=15. *Eight years*: event n=19, censored n=70, missing n=61, censored cases before the earliest event in a stratum n=8. *Nine years*: event n=24, censored n=71, missing n=63
Reference category is no overweight or obesity

Supplementary Table S12

BMI Status at Ages Five to Nine Years of as a Predictor of FVC <1L Using a Cox Proportional Hazards Model [1]

Age at BMI measure	BMI status	Hazard Ratio	95% CI lower	95% CI upper	P-value
**Six years**	*Overweight*	1.046	0.270	4.045	0.948
	Obesity	0.919	0.243	3.480	0.901
**Seven years**	*Overweight*	1.194	0.412	3.458	0.744
	Obesity	0.762	0.202	2.881	0.689
**Eight years**	*Overweight*	0.623	0.113	3.429	0.586
	Obesity	2.716	0.773	9.547	0.119

[1] *Five* and *eight years*: Not able to be performed as coefficients did not converge. *Six years*: event n=14, censored n=59, missing n=85. *Seven years*: event n=17, censored n=71, missing n=70. *Nine years*: event n=13, censored n=62, missing n=72, censored cases before the earliest event in a stratum n=11
Reference category is no overweight or obesity

Supplementary Table S13

Predictors of Obesity Using a Generalized Estimating Equation (N=138 Patients, N=2246 Observations)

Predictors [1]	OR	95% CI lower	95% CI upper	P-value [2]
(Intercept)	0.918	0.618	1.363	0.671
Age	0.991	0.971	1.011	0.392
Length of follow-up	0.994	0.971	1.018	0.642
Age at diagnosis	1.012	0.968	1.059	0.591
Ambulant [a]	0.921	0.806	1.054	0.231
≥7 seconds in a 10m walk/run [b]	1.132	0.933	1.375	0.209
10m walk/run data missing [b]	1.153	0.958	1.387	0.132
≥ 1 fracture [c]	1.103	0.955	1.273	0.182
≥ 1 dietitian consult [d]	1.188	1.064	1.327	**0.002**
Scoliosis surgery (yes) [e]	0.449	0.351	0.574	**<0.001**
Steroid-treated predisolone only [f]	1.090	0.978	1.215	0.120
Steroid-treated prednisolone then deflazacort [f]	1.298	1.093	1.543	**0.003**
Steroid-treated vamorolone [f]	1.297	0.445	3.774	0.634
Dystrophin isoforms maintained: Dp140, Dp116 and Dp71 (Category 2) [g]	0.776	0.577	1.043	0.093
Dystrophin isoforms maintained: Dp116 and Dp71 (category 3) [g]	0.941	0.789	1.123	0.501
Dystrophin isoforms maintained: Dp71 OR nothing maintained (Category 4 or 5) [g]	0.830	0.583	1.182	0.302
Unknown/missing genetic information [g]	0.782	0.645	0.948	**0.012**
Diagnosis of a neurodevelopmental disability [h]	0.826	0.695	0.983	**0.031**

1 Reference categories are: [a] Non-ambulant [b] Less than 7 second [c] No fracture [d] No dietitian consult [e] No scoliosis surgery [f] Steroid-naïve [g] Dp260, Dp140, Dp116 and Dp71 maintained [h] No diagnosis of neurodevelopmental disability

2 P-values in boldface indicate statistical significance using a p-value of <0.05
